# Supplementary material for: Chromothripsis during telomere crisis is independent of NHEJ, and consistent with a replicative origin
Source: Genome Res. 2019 May;29(5):737–49. doi: 10.1101/gr.240705.118 (PMC6499312; doi:10.1101/gr.240705.118)
Supplement: Supplemental Material [file supp_gr.240705.118_Supplemental_file_1.zip › contigs/annotated_contigs/DB73/contig.2.DB73_length_427_mean_cov_7.29274004684.docx]

**DB73_length_427_mean_cov_7.29274004684**

```
TTGCTCTGTCACCCAGGCTGGACTGCAATGACACAATCTCAGCTCACCGCAGCCTTCACCTCTCAGGTTCAAGAGATTCTCCTGCCTCA
 >chr3:82289321-82289672 - E=5e-197
GCATCCCAAGTAGCTGGGATTACAGATGTGCACCACCATGCCTGGCTAATTTTTGCATTTTTAGTAGAGATGGGTTTTCGCCATGTCGG

CCAGGCTGGTCTTGAGCTCCTGAGCTCAAGTGATCTACCCGCCTCTGCCTCCCAAAGTGCTGGGATTACCAGTGTGAGCCACAGCACCC

AGCCGAGTCCTGCCCTTTTTGCTGTCTATTTAAATGCTATATTTTACCCATTTTGTGCTTTTAGTTGGTGATTTTGCTGTTTA|A|TGT
                                                                                    >ch
AAGCCAATTTATTATAATAAATCTCTCTCTGTGTCTGTCTGGATATTGATATATTATTGAATTTTCCAAAATTr3:81208171-81208248 - E=8e-35
```
